# A Novel Algorithm to Enhance P300 in Single Trials: Application to Lie Detection Using F-Score and SVM

**DOI:** 10.1371/journal.pone.0109700

**Published:** 2014-11-03

**Authors:** Junfeng Gao, Hongjun Tian, Yong Yang, Xiaolin Yu, Chenhong Li, Nini Rao

**Affiliations:** 1 College of Biomedical Engineering, South-Central University for Nationalities, Wuhan, People's Republic of China; 2 Nanjing Fullshare Superconducting Technology Co., Ltd., Nanjing, People's Republic of China; 3 School of Information Technology, Jiangxi University of Finance and Economics, Nanchang, People's Republic of China; 4 Department of Information Engineering, Officers College of CAPF, People's Republic of China; 5 School of Life Science and Technology, University of Electronic Science and Technology of China, Chengdu, People's Republic of China; University of Ulm, Germany

## Abstract

The investigation of lie detection methods based on P300 potentials has drawn much interest in recent years. We presented a novel algorithm to enhance signal-to-noise ratio (SNR) of P300 and applied it in lie detection to increase the classification accuracy. Thirty-four subjects were divided randomly into guilty and innocent groups, and the EEG signals on 14 electrodes were recorded. A novel spatial denoising algorithm (SDA) was proposed to reconstruct the P300 with a high SNR based on independent component analysis. The differences between the proposed method and our/other early published methods mainly lie in the extraction and feature selection method of P300. Three groups of features were extracted from the denoised waves; then, the optimal features were selected by the F-score method. Selected feature samples were finally fed into three classical classifiers to make a performance comparison. The optimal parameter values in the SDA and the classifiers were tuned using a grid-searching training procedure with cross-validation. The support vector machine (SVM) approach was adopted to combine with an F-score because this approach had the best performance. The presented model F-score_SVM reaches a significantly higher classification accuracy for P300 (specificity of 96.05%) and non-P300 (sensitivity of 96.11%) compared with the results obtained without using SDA and compared with the results obtained by other classification models. Moreover, a higher individual diagnosis rate can be obtained compared with previous methods, and the presented method requires only a small number of stimuli in the real testing application.

## Introduction

Research into lie detection has drawn a substantial amount of attention over the past several decades and has found many important applications in the legal, moral and clinical fields [1]–[3]. Currently, a number of studies that adopt neurophysiological signals have been conducted on lie detection. These methods have used Magnetic Resonance Imaging [4], [5] and Event-Related Potentials (ERPs) [6], [7]. P300, an endogenous ERP component, has been extensively investigated [8] and has been successfully used for deception detection [9].

Widely used P300-based lie detection methods can be roughly divided into three categories: the bootstrapped amplitude difference (BAD) [10], [11], the bootstrapped correlation difference (BCD) [12] and machine learning methods [7], [13], [14]. For the methods listed above, there are three types of stimuli that are presented to subjects, i.e., Probe (P), Target (T) and Irrelevant (I) stimuli [7].

A good lie detection method should use a small number of stimuli to achieve as high accuracy as possible. To realize this goal for the P300-based lie detection, a critical step is to extract the P300 with a high signal/noise ratio (SNR). Although the P300 is time- and phase-locked to experimental stimuli, the extraction of the P300 with a high SNR is still a challenging task because various types of noise are superimposed seriously on P300 [15]. BAD and BCD use the statistical technique of bootstrapping [16] to generate many different averages of ERP from the same set of stimuli [7]. Using bootstrapping, the SNR of P300 can be increased. However, such a mode involves a large number of stimuli and hence is at the expense of taking a longer time for signal acquisition, which would also increase the fatigue of the subjects. In addition, more recently, a few researchers have investigated single trial-based lie detection methods that were based on machine learning [7], [14]. In these methods, some features were extracted from single trials and then were used to train classifiers to differentiate between different brain states. The testing results showed that machine learning methods could achieve a higher detection accuracy than BAD and BCD methods [7]. However, they typically did not remove the noises embedded in single trials, resulting in unsatisfactory detection accuracy.

Consider the noises embedded in single trials for P300 extraction. The EEG recording on one sensor consists of two main parts. One part is extra-skull noise, and the other part is the signal produced by intra-skull neuronal sources at specific brain regions, including ERP and spontaneous EEG. Obviously, the ERP cannot be represented by the signal from the sensor directly. Conventional lie detection methods could not separate P300 from the noise and spontaneous EEG because their time courses and scalp projections usually overlap [17]. Recently, independent component analysis (ICA), a blind source separation (BSS) method [15], [18]–[20], was used to extract stimulus-related ERP into independent components (ICs) [21]–[24]. The results showed that the decomposed ICs were more distinguishable than the “sensor signals” [22], [23]. In our early study [25], we proposed an ICA-based template matching method, topography-template matching (TTM) algorithm, to enhance the SNR of P300, and we achieved promising results. In TTM, we only consider the P300 independent sources affect in Pz site. In addition, one neurophysiologist was employed to select the P300 independent source by his experience. In this study we present a novel spatial denoising algorithm (SDA) to improve that early study. Comparing with our early study, SDA consider more affecting areas including at P3, P4, Pz, Cz and Oz sites. In addition, SDA recognized P300 independent source automatically, not by experience. Hence, the SDA is more reasonable and objective than the early study. The key innovation is how to automatically identify the P300 ICs (i.e., the ICs accounting for the P300), which will be described in the following section.

By removing any redundant features, feature selection can help the original classification system to achieve better classification performance including lower computational costs and higher classification accuracy. Polat et al. indicated that feature selection improves the classification accuracy by using a hybrid system of feature selection and several classifiers [26]. In this study, the F-score [38], a simple but effective technique, was used to select the optimal features from the original extracted features. In addition, to select a suitable classifier, all of the training samples with the selected optimal features were fed into three popular classifiers to compare their performance.

For conventional lie detection like BCD/BAD [10]–[12] and other some lie detection methods [7], [13], a number of stimuli were required to present to the subjects in practical applications, because both of the bootstrapping technique and threshold selection-based classification were based on many stimuli responses. This would limit the real application of lie detection. First, there is often very limited information related to criminal acts. Second, many repeated stimuli with little information would cause two problems. One problem is fatigue, and the other is an increase in the countermeasures [11], because real criminals might be familiar with the stimuli and tend to resist the detection when many stimuli are presented repeatedly. Furthermore, based on the analysis results from a number of stimuli, when the researcher need to make the last judgment, a threshold strategy (see the references [10]–[12], [7], [13] for details) was inevitably used, which was a subjective decision on the individual diagnostic rate. The present method aims at using only a small number of stimuli and having no threshold problem.

## Materials

### Ethics statement

The experiment was approved by Psychology Research Ethical Committee (PREC) of the College of Biomedical Engineering in South-Central University for Nationalities. Thirty healthy subjects (15 females, mean age of 21.5) were recruited from the university. The participants provided their written informed consent according to a human research protocol in this study.

### EEG Data Acquisition

Twelve electrodes (Fp1, Fp2, F3, Fz, F4, C3, Cz, C4, P3, Pz, P4, Oz) from an International 10–20 system were used. The vertical EOG (VEOG) signal was recorded from the right eye (2.5 cm below and above the pupil), and the horizontal EOG (HEOG) signal was recorded from the outer canthus. EEG and EOG signals were filtered online with a band pass filter of 0.1–30 Hz, and they were digitized at 500 Hz using Neuroscan Synamps. All of the electrodes were referenced to the right earlobe. Electrode impedances did not exceed 2 k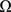.

### Experimental Protocol

The standard three-stimuli protocol [10], [12] was employed in this study. The participants were randomly divided into two groups: a guilty group and an innocent group. Six different jewels were prepared, and their pictures served as stimuli during detection. A safe that contained one (for the innocent) or two (for the guilty) jewels was given to each participant. They were instructed to open the safe and memorize the details of the object. We instructed the guilty group to steal only one object which would serve as the P stimulus. The other object in the safe was the T stimulus, and the remaining four pictures were the I stimuli. The object in the safe was not stolen for the innocent, which served as the T stimulus. Then, from the remaining five pictures, one picture was selected randomly and set as the P stimulus, and the remaining four images were set as I stimuli. All of the subjects were instructed to write down the information on the objects in the safe, such as the styles and colors of the jewels.

**Figure 1:**
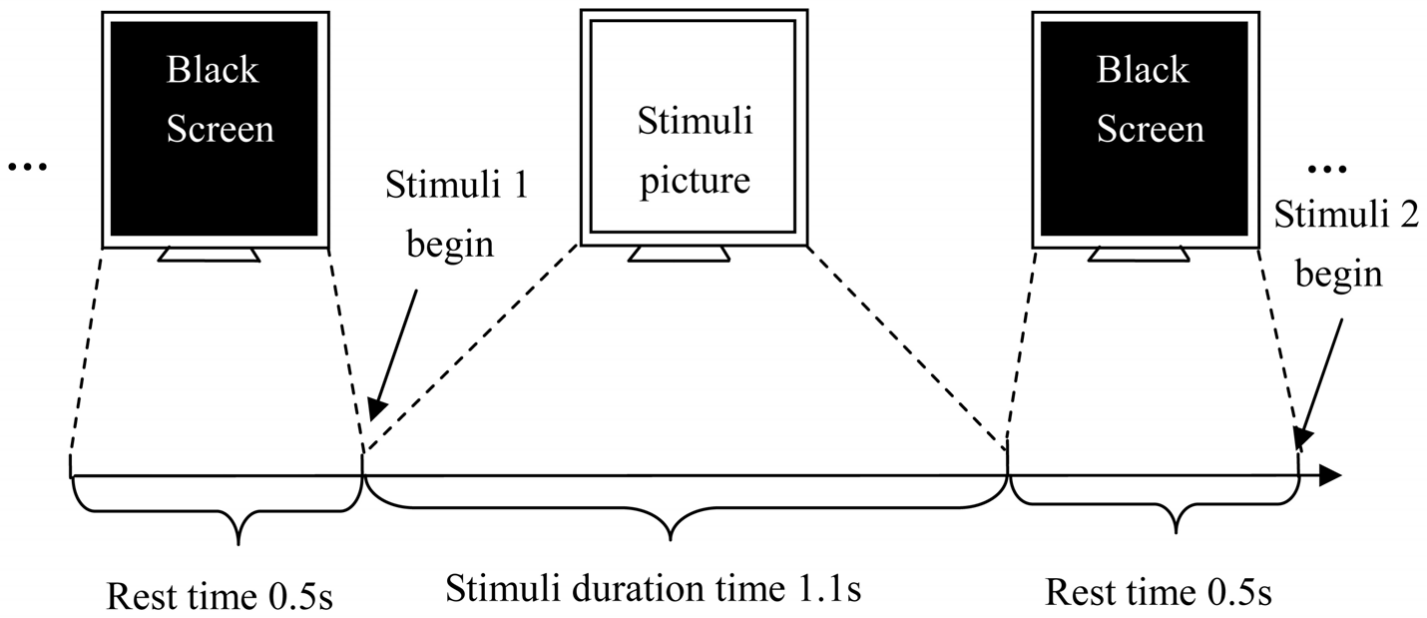
The stimuli sequence diagram.

After the preparation tasks introduced above, the participants began to perform the detection. They were seated in a chair, facing a video screen that was approximately 1 m away from their eyes. The stimuli pictures were presented randomly on the screen. Each item remained for 0.5 s with 30 iterations for one session, and each session lasted for approximately 5 minutes, with 2 minutes of resting time. The inter-stimulus interval was 1.6 s. Each subject was instructed to perform 5 sessions. The stimuli sequence diagram is given in Figure 1. One push button was given to each subject, and he or she was asked to press a “Yes” and “No” button when faced with familiar and unknown items, respectively.

**Figure 2:**
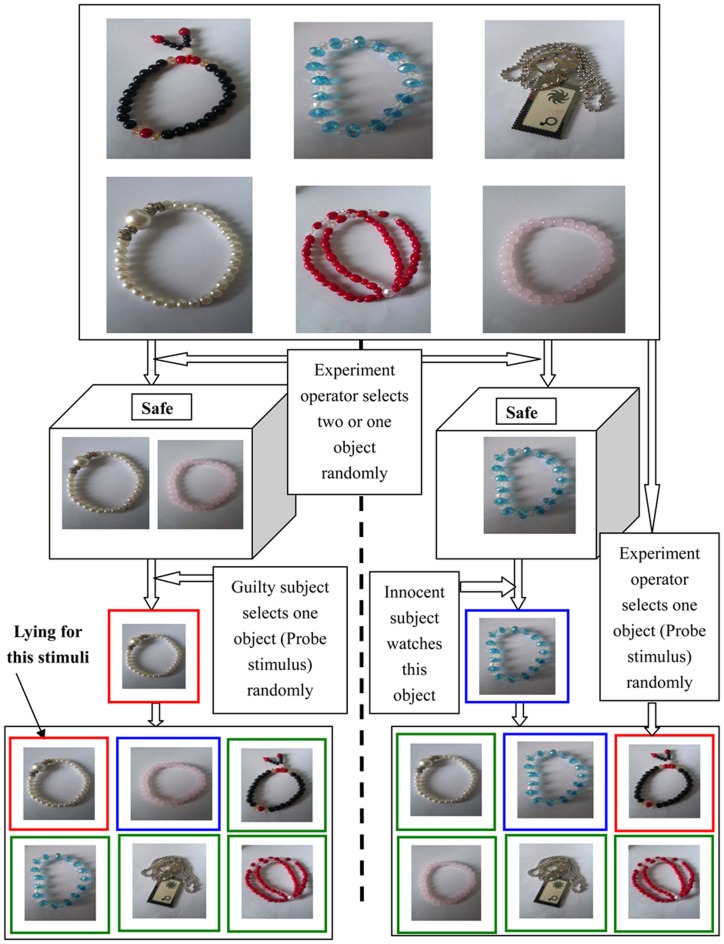
The sketch map of stimuli protocol. The left part and right parts of the dashed line represent the experimental protocol for guilty and innocent subjects, respectively. The pictures with red, blue and green rectangles represents P, T and I stimuli, respectively.

The guilty group was instructed to press the “Yes” and “No” button when faced with the T and I stimuli, respectively. With a P stimulus, they were asked to press the “No” button, attempting to hide the stolen act. In contrast, the innocent group made honest responses to all of the stimuli. All of the subjects had practiced the tasks above before the EEG signals were recorded formally. We planned to exclude any subjects that had more than a 5% clicking error, but none fell into this category. Finally, a sketch map is presented and shown in Figure 2 to describe above protocol.

## Methods

### General description of method

**Figure 3:**
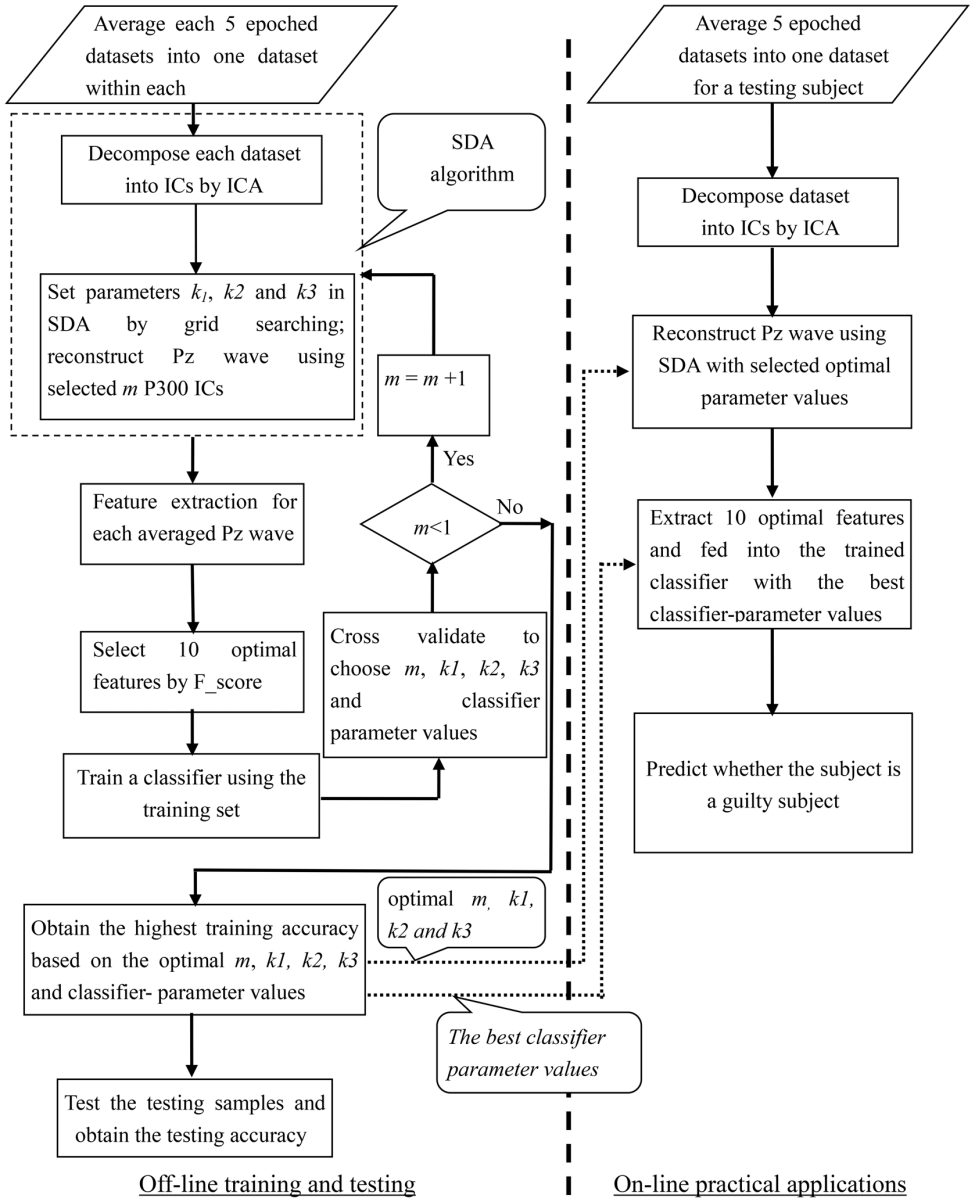
The flowchart of the presented CIT system.

The present method is separated into the following steps: (1) preprocess the continuous raw EEG recordings, and then, apply SDA on the preprocessed datasets to reconstruct P300 waves that have a higher SNR (from the guilty) and non-P300 waves (from the innocent). For convenience, we hereafter describe the above processed results as reconstructed P300 waves (In fact, the results also contain non-P300 waves); (2) extract original features from the reconstructed waves; (3) adopt the F-score method to select the optimal features; these features were concatenated as a featured vector and fed into three kinds of typical classifiers; (4) train the classifiers using the two classes of training samples, and then, test the samples using testing samples. By the training procedure, the optimal parameter values including the parameter in SDA and in specific classifier can be determined. During a practical application phase, only several stimuli (Five probe stimuli were needed in this study) are presented to the subjects. The flowchart of the presented CIT system is shown in Figure 3.

### Preprocessing

Using EEGLAB toolbox, we segmented the continuous EEG data into epoched datasets, each of which lasted from 0.5 s before to 1.1 s after the stimulus onset. Then, the ocular artifacts [24] in each set were removed by the software SCAN of Neuroscan, i.e., the datasets that contained single trials with the voltage in excess of 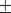
75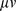 were discarded. All of the remaining trials were baseline corrected on the pre-stimulus interval. Lastly, the datasets corresponding to P responses were selected, and each 5 datasets within each subject was pooled into one average, resulting in 450 averaged datasets for each subject group.

### Independent component analysis

Let **X**(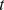) = 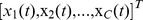
denote the observed time series with 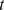 varying from 1 to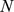, where 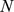 and 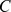
denote the number of samples and sensors, respectively. In ICA method, **X**(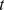) is the result of an unknown mixture of a set of unknown source signals **S**(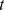)  = 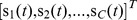, and the mixture is viewed as linear: **X**(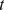)  = **AS**(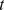). Based on the principle of statistical independence [26]–[27], ICA estimates **S**(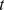) by introducing the unmixing matrix **W**, i.e., **Z**(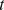)  = **WX**(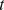) where **Z**(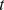) (which is the decomposed ICs) is the estimation of signals **S**(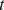). Accordingly, 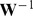 is referred to as a mixing matrix. Once the signals **S**(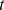) are estimated by an ICA algorithm, a column of the matrix 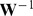 provides the projection strengths of the corresponding IC onto each electrode.

### Spatial denoising algorithm for P300 enhancement

The spatial denoising algorithm, referred to as SDA hereafter, is described in this section. First, each averaged dataset was decomposed by ICA, resulting in mixing matrix 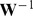 and decomposed ICs **Z**(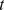). The extended infomax algorithm (EICA) was used in ICA because it can allow some sources to have sub-Gaussian distributions [28], [29]. By accommodating sub-Gaussian distributions in the data, EICA could provide a more accurate decomposition of multi-channel EEG signals, especially when various neurophysiological signals follow different distributions.

Many investigators have found that P300 was usually the largest at Pz, the smallest at Fz, and takes intermediate values at Cz [30], [32]. They typically acquired the P300 on one of the electrodes listed above [7], [9], [11], [31]. According to the *a priori* physiological knowledge described above and the spatial distribution of an IC, SDA is divided into the following four steps:

1. Let 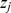 denote the *j*th IC in matrix **Z**(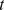). Denote the *i*th row *j*th column element in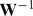 by 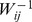, and accordingly the *j*th column by 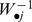. First, each matrix
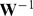
is normalized to the matrix 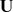 by

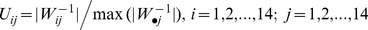
(1)where symbol 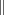 denotes an absolute calculation. Let 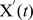 denote a new EEG dataset, which was defined by
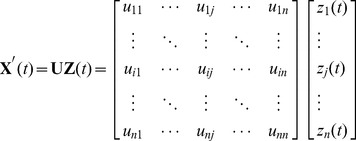
(2)
2. Let *Pz*, *P3, P4, Cz* and *Oz* equal their respective sequence number in the electrode set (e.g., *Pz* equals 10 in this study). For the *j*th column in each matrix **U**, we calculate a value 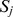 using the following formula:

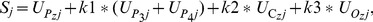
(3)where the parameters *k1, k2* and *k3* denote the weighted parameters on different element 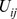. A grid-search procedure (see Figure 3) would be used to obtain optimal values of these parameters. In this equation, 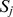
denote the integrated distribution-strength on several interested brain areas from *j*th IC. The bigger 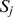 is, the bigger probability *j*th IC is the P300 ICs.
3. Sort the 14 values in 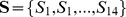 in descending order, resulting in a sorted vector **E** and a sorted index vector 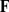, with 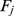 being the position of the element in vector **S**.
4. Back projection: Let *m* denote how many P300 ICs should be selected to reconstruct the P300 wave. Suppose that 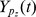 is the reconstructed P300 wave on the Pz electrode. The procedure of back projection for 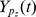 can be given by

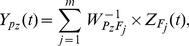
(4)i.e., only *m* ICs are considered as P300 ICs and are back projected to the scalp.

A grid-search procedure (see Figure 3) will be used to determine the optimal value of parameter *m*, which will be discussed later.

Lastly, for two groups of subjects, two sets of the reconstructed waves can be obtained, respectively. Let **R–G** denote the vector set for the guilty group, and let **R–I** denote for the innocent group. We expect that the SNR of P300 in the set **R–G** would be enhanced compared with the raw ERP signal, using the above SDA.

### Feature extraction

Let 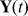 denote a time wave in the set **R–G** or **R–I**, with *t* varying from stimulus onset to 1.1 s after the stimulus onset. Time-domain, frequency-domain and wavelet features were selected as three groups of features in this study. Most of them have been demonstrated to be effective by many researchers [7], [25], [33]–[35]. The features are extracted from each signal 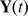 by the following procedure.

#### Time-domain features

Four time-domain features are defined as follows:

1. Maximum amplitude, which is defined as
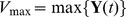
(5)
2. Latency, which is the time where 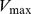 occurs. It takes the form
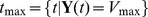
(6)
3. Peak-to-Peak, which is defined as
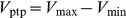
(7)
4. Positive area, which is the sum of the positive signal values. It can be expressed as
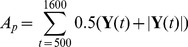
(8)

#### Frequency-domain features.

The power spectrum density (PSD) is *first* calculated on each 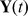 by the Bartlett algorithm. Let 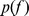 be the resultant PSD. Suppose that 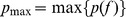 denotes the maximum amplitude value of the PSD. Then 3 frequency-domain features can be calculated as follows:

1. Maximum frequency, i.e.,
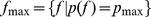
(9)
2. Mean frequency, calculated by the weighted average of the frequency. The weighted coefficient is the PSD value. It can be expressed as
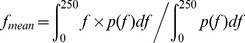
(10)
3. The power of the main frequency band that involves the P300, which is calculated by

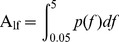
(11)

#### Wavelet features

Many authors have indicated that ERPs are transient signals that include some typical frequency components in a different frequency range, such as delta, theta, alpha, beta and gamma [36]. Recently, the wavelet transform (WT) has been widely used to analyze ERPs [36]–[38]. The WT is achieved by the breaking up of a signal into shifted and scaled versions of the mother wavelet, which is a waveform that has a limited duration and a zero mean.

In this study, a fast algorithm for the Discrete WT (DWT) was adopted to decompose those averaged single trials [39]. We selected Quadratic B-Spline functions as mother wavelets because they have a near-optimal time-frequency localization property and good similarity with the P300 components [40]–[41]. The wavelet coefficients were computed by a high-pass filter **h** and a low-pass filter **g**. The coefficients of two filters are given in the first and second columns of Table 1, respectively. The reconstruction filters **H** and **G** can be used to inversely transform the wavelet coefficients to time-domain waveforms. The third and fourth columns of Table 1 give the coefficients of the two reconstruction filters, respectively.

**Table 1:** Coefficients of the truncated decomposition filters *h*, *g* (IIR) and reconstruction filters H, G (FIR) for quadratic spline filters.

*e*	*h(e)*	*g(e)*	*H(e)*	*G(e)*
−10	+0.00157	−0.00388		
−9	+0.01909	−0.03416		
−8	−0.00503	+0.00901		
−7	−0.04440	+0.07933		
−6	+0.01165	−0.02096		
−5	+0.10328	−0.18408		
−4	−0.02593	+0.04977		+1/480
−3	−0.24373	+0.42390		−29/480
−2	+0.03398	−0.14034	0.25	+147/480
−1	+0.65523	−0.90044	0.75	−303/480
0	+0.65523	+0.90044	0.75	+303/480
1	+0.03398	+0.14034	0.25	−147/480
2	−0.24373	−0.42390		+29/480
3	−0.02593	−0.04977		−1/480
4	+0.10328	+0.18408		
5	+0.01165	+0.02096		
6	−0.04440	−0.07933		
7	−0.00503	−0.00901		
8	+0.01909	+0.03416		
9	+0.00157	+0.00388		

DWT was performed on each wave 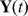, which resulted in seven sets of wavelet coefficients corresponding to different frequency bands: 0.3–3.9, 3.9–7.8, 7.8–15.6, 15.6–31.2, 31.2–62.5, 62.5–125 and 125–250 Hz. Only the first four bands were useful due to the earlier filtering. Because the *delta* band was the main frequency range for the P300 component, the coefficient set corresponding to the first frequency band was selected as the final wavelet features for each wave 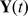.

Following the feature extraction, these feature samples were divided into two sample sets: the first set contained all of the *P300 samples* for the guilty group, and the second set contained *non-P300 samples* for the innocent group, with the class label being 1 and −1, respectively.

### Feature Selection

In this study, we adopted the F-score method to further select the best subset of features for classification. The F-score method is a very simple but robust feature-evaluating technique. Recently, many researchers have successfully used this method in pattern recognition systems to select the optimal feature subset [42], [43].

Given the *i*th feature vector 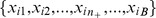 with the number of positive instances *n_+_* and the number of all of the instances *B*, the *F-score* value of the *i*th feature is defined by



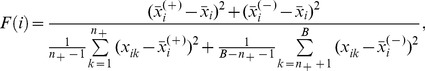
(12)


where 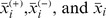 are the average of the positive, negative, and whole samples, respectively, and 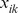 is the *k*th feature value in the *i*th feature vector. Positive and negative represent two classes of identification, respectively. A larger *F-score* value indicates that the feature has more discriminative power. For the application of this method, the *F-score* value of all of the features will be sorted. Hence, in this study, those features that have relatively larger F-score values were selected to construct the feature subset.

There are two main methods used to select the appropriate feature subset: the filter method [44] and the wrapper method [45], [46]. To obtain simplicity and a lower computation cost, we used the former method to select the feature number for the optimal feature subset.

### Classification

The fisher discriminant analysis (FDA) [47], back propagation neural network (BPNN) [48] and support vector machine (SVM) [49], [50] were compared in this study to select an optimal classifier. The details of the three classifiers are given in Supporting information files (see Section S1–S3 in File S1). The hybrid models integrating with F-score feature selection is referred to as F-score_FDA, F-score_BPNN and F-score_SVM in this study. Accordingly, three individual classification models (FDA, BPNN and SVM) were also utilized.

A Subject-Wise CV (SWCV) [25], [51] was performed on the two classes of optimal feature sample sets. For each set, samples from 14 subjects were grouped into a training set and the samples from the remaining were used as a testing set. Thus by this SWCV, 15 pairs of training sets and testing sets were obtained. For each pair, the training set consisted of the samples from 28 subjects, and the testing set from 2 subjects (i.e., a guilty and an innocent subject). We would like to emphasize the importance of the SWCV procedure. In fact, a statistical classification model that could explain the data for some subjects did not necessarily generalize well to other subjects, even if those were draw from the same distribution. Accordingly, the SWCV procedure was used to assess the generalization ability not only from the different data within one subject but from the data in different subjects. Hence, the advantage of SWCV compared with common CV is that the test accuracy can simulate the generalization performance on other unseen subjects. Accordingly, we can obtain the testing results not only on the level of single-trials, but also on the level of subjects, i.e., to test whether one subject can be recognized correctly.

**Figure 4:**
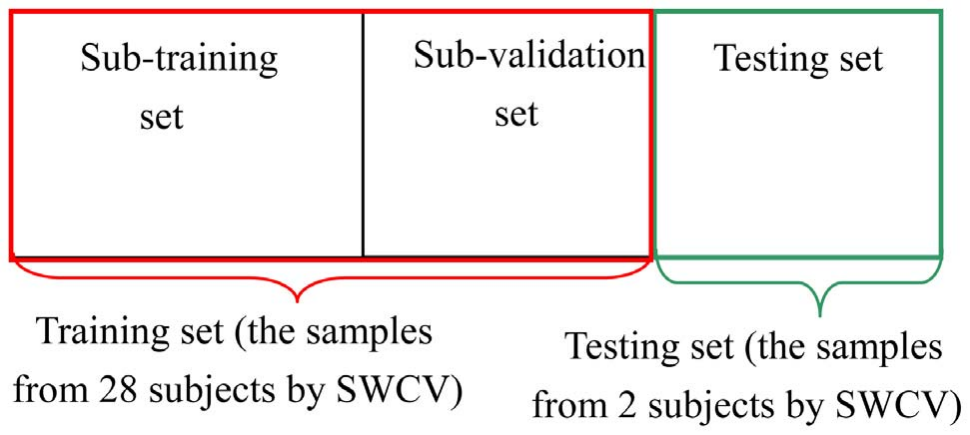
The division of feature samples using SWCV and 10-fold CV. The red rectangle denotes training set, whereas the green rectangle denotes testing set by the division of SWCV; Training set is further divided into sub-training set and sub-validation set by common 10-fold CV.

For each training set yielding by SWCV, the feature samples were mixed to obtain two classes of samples: one is lying group (it was considered as P300 feature samples) and the other is truth-telling group (it was considered as non-P300 feature samples). Subsequently, a common 10-fold CV procedure [52] was performed on each training set, resulting in 10 pairs of sub-training sets and sub-validation sets. Figure 4 shows the schematic diagram of the division of samples and cross validation procedure.

### Selection of optimal parameters

For the proposed lie detection method, two groups of parameters must be tuned: 1) The parameters in SDA: *m*, *k1*, *k2* and *k3*, and 2) The specific hyperparameters for each classifier. Considering that the parameters in SDA can affect the optimal values of the hyperparameters, the two groups of parameters were tuned together using a multi-dimension grid searching. During the turning, *m varied* from 1 to 14; and *k1*, *k2* and *k3* varied from 0.2 to 1 with a step size of 0.15, by the suggestion of an independent EEG expert. In the tuning procedure above, for BPNN, the number of sigmoid hidden nodes 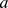 and the learning rate 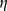 were tuned (the control precision was set to be 0.002). For SVM, the penalty parameter *C* and the radial width 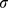
for radial basis function (RBF) (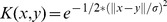, [52]) were tuned. The procedure of training and testing is described as follows:

1. The classifiers were trained on each sub-training set with different combinations of tuning parameters. By the 10-fold CV, an averaged sensitivity and an averaged specificity can be obtained for the *j*th training set. Then, the *mean* and *Standard Deviation* (*SD*) of the 15 sensitivities (15 training sets), referred to as 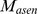 and 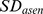 respectively, are calculated. Similarly, the 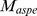 and 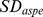 for specificity are obtained. Lastly, *balanced accuracy* 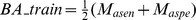 is calculated for the specific combination of tuning parameters.
2. Repeat the above steps using a different combination of tuning parameters. Thus, the optimal parameter values were selected when 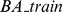 reached the highest value.
3. On the 15 testing sets, calculate the generalization performance of the trained classifiers with the optimal parameter values. Similar to step 1, 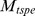 and 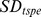 (*mean* and *SD* on the 15 sensitivities), 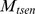 and 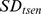 (on the 15 sensitivities) can be obtained. Finally, calculate the balanced testing accuracy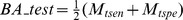. This accuracy is the final testing measure of the performance evaluation.

## Results

### Preprocessing

**Figure 5:**
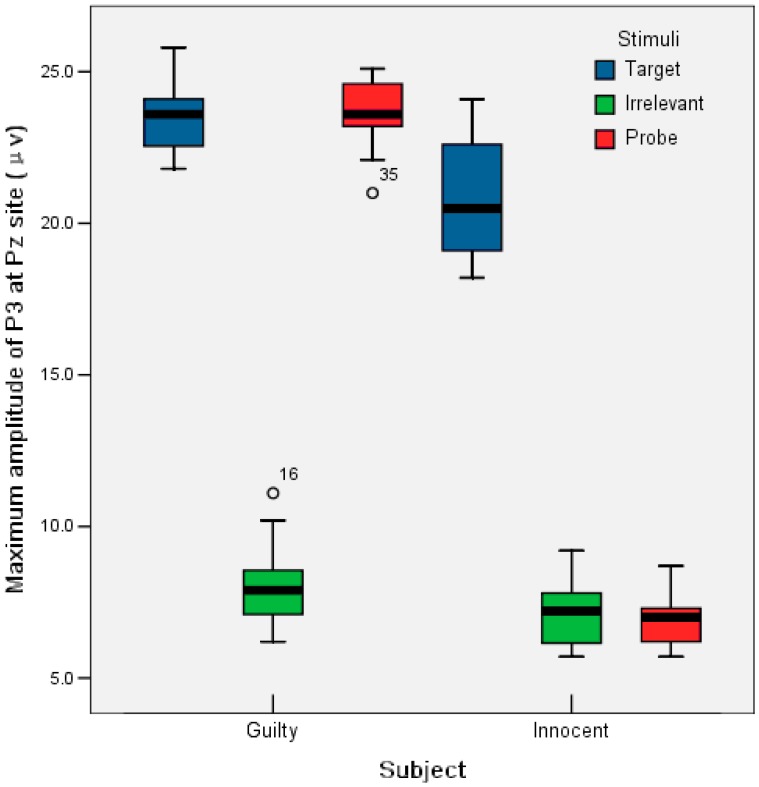
Boxplot of the maximum amplitude of P300 at Pz in different stimuli and subject groups.

The grand average ERPs on the Fz, Cz, Pz and Oz sites as a function of stimulus type were first calculated within each subject. Figure 5 gives the boxplot of the maximum amplitude at the Pz site for three types of stimuli and the two subject groups, during which 450 samples for each type of stimuli and each group were used to statistical analysis. Using ANOVA on the guilty subject, there is no significant difference (*p*>0.05) for the maximum amplitude between the P and T stimuli. However, there is a significant difference (*p*<0.001) between P and I stimuli. In contrast, there is no significant difference (*p*>0.05) between the P and I stimuli for an innocent subject.

**Figure 6:**
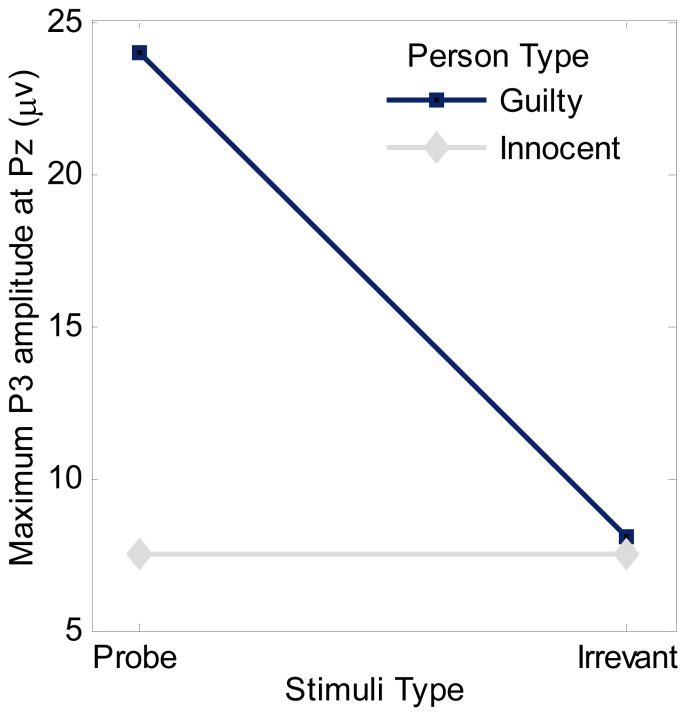
Maximum P300 amplitude at Pz as functions of person type (guilty and innocent) and stimuli type.

A 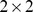 mixed model ANOVA (P vs. I 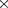 innocent vs. guilty) was performed on the maximum amplitude at the Pz site. The result shown in Figure 6 revealed significant main effect of innocent versus guilty, *F*(1, 28) = 772.467, *p*<.0005 and P versus I, *F*(1, 28) = 761.201, *p*<.005. There is also significant interaction between innocent versus guilty and P versus I, *F*(1, 28) = 753.430, *p*<.005.

More importantly, by a further independent effect analysis of innocent versus guilty when P stimuli was used, the person type effect is significant and yields *F*(1,28) = 1514.68, *p*<.0005. The amplitude of P300 for the guilty is higher than that for the innocent. In contrast, when using I stimuli, there is no significant person effect (*F*<1). Hence, P responses at the Pz site were finally selected for further processing to enhance the feature difference of the P300 waves between the two classes of subjects.

### SDA

**Figure 7:**
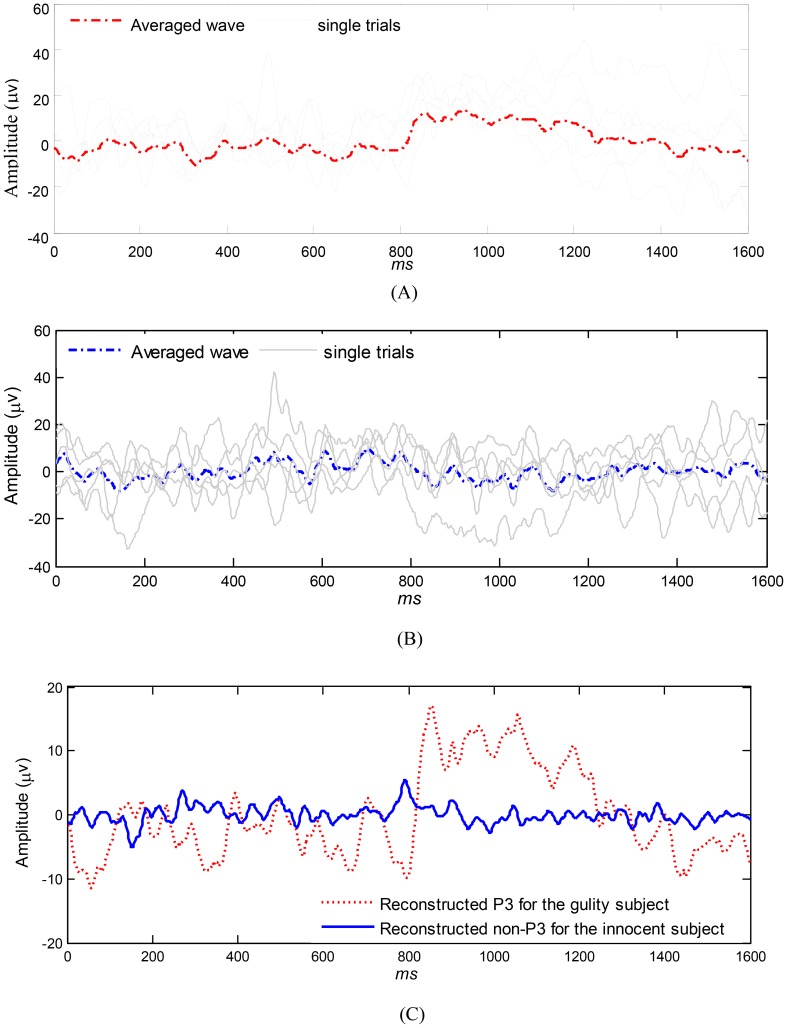
Response waveforms and reconstructed waveforms on Pz after applying SDA for a guilty and an innocent subject. 7A: Single trials (solid lines) and averaged waveform (dashed line) on Pz for a guilty subject before applying SDA. 7B: Single trials (solid lines) and averaged waveform (dashed line) on Pz for a guilty subject before applying SDA. 7C: Reconstructed waveforms (a P300 for the guilty subject and a non-P300 for the innocent subject) by applying SDA on the averaged datasets.

First, the enhancement of the SNR of P300 by SDA is illustrated in Figure 7. A guilty subject's five raw EEG datasets were randomly taken as an example. The raw waves on the Pz with solid thin line and their averaged wave with dashed thick lines are shown in Figure 7A. Similarly, we randomly selected an innocent subject, and the raw waves and averaged wave on Pz are shown in Figure 7B. Applying SDA to the two averaged datasets respectively, the two reconstructed P300 waveforms on Pz are shown in Figure 7C. There is no distinct P300 (dashed lines) in Figure 7A and 7B. As Figure 7C shows, however, there is a clear P300 with a latency of approximately 280 ms for the guilty subject, and the two lines can be differentiated easily. During this evaluation, the parameters *m*, *k1*, *k2* and *k3* were set to 3, 0.9, 0.8, 0.6 by *a priori* knowledge of an independent physiology expert.

### Extraction of Wavelet Features

**Figure 8:**
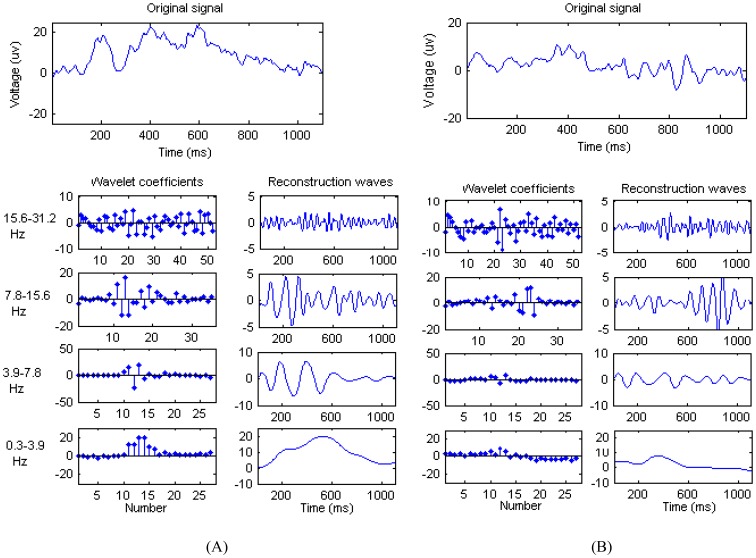
The wavelet coefficients in 4 bands and corresponding reconstructed waveforms. 8A: The original EEG waveforms on Pz for a guilty subject (above panel), its wavelet coefficients (left column) and corresponding reconstruction waves (right column). 8B: The original EEG waveforms on Pz for an innocent subject (above panel), its wavelet coefficients (left column) and corresponding reconstruction waves (right column).

After SDA, the features were extracted from the reconstructed waves for the Pz. Here, we randomly selected a guilty and an innocent subject, and then conducted the wavelet transform on two subjects' denoised P300 signals, respectively. The results of DWT are shown in Figure 8A and 8B respectively. The most distinct difference in the wavelet features and reconstruction waves between the two subjects is in the 0.3–3.9 Hz band (the delta band). For the guilty subject, it can be seen from the bottom row in Figure 8A that there are obvious peaks in the wavelet coefficients and reconstruction waves at approximately 500 ms post-stimulus for this band. This approach is in accordance with the time-domain features of the P300 waveform. In contrast, there are no obviously corresponding features in Figure 8B. The results above suggest that the wavelet coefficients corresponding to the delta band, as a class of P300 features, are suitable for differentiating the P responses between the two groups of subjects.

### Result of the feature selection

Table 2 shows the results of the feature selection by the F-score method. *W_1_*–*W_22_* denotes 22 WT coefficients. From this table, we can see the *F-score* values of the 29 original features. Those features with relatively larger *F-score* values were selected to construct a feature subset. For simplicity, we directly selected 10 features whose *F-score* values were larger than 0.85 to form the optimal feature subset.

**Table 2:** The results of feature selection on original 29 features using F-score.

Features	*F-score* values
*V*_max_	0.937
*t*_max_	0.567
*V*_ptp_	0.877
*A*_p_	0.268
*f*_max_	0.049
*f*_mean_	0.340
*A*_lf_	0.873
*W*_1_–*W*_5_	0.085, 0.005, 0.311, 0.011, 0.099
*W*_6_–*W*_10_	0.008, 0.184, 0.106, 0.077, 0.381
*W*_11_–*W*_16_	0.977, 0.524, 0.255, 0.835, 0.820, 0.947
*W*_17_–*W*_22_	0.905, 0.937, 0.881, 0.959, 0.871, 0.838

Observing these 10 features, we can see that two optimal time-domain features are closely related to the peak value of P300. Second, one feature (A_lf_) is related to the main frequency range of P300 (0.3–3.9 Hz). Most importantly, the most of optimal features are selected from the original wavelet features. This indicates the wavelet feature has the better classification capability than the other two kinds of features.

### Classification Performance

**Figure 9:**
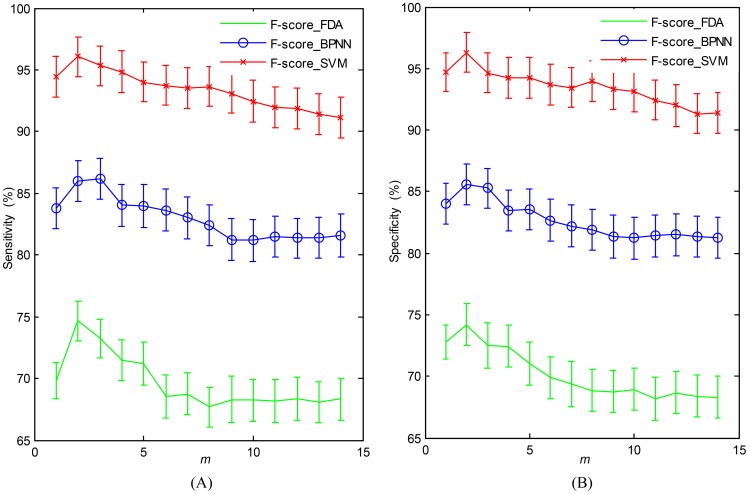
The accuracy (mean 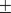
 **SD) of classifying P300 (sensitivity) and non-P300 (specificity) for three classification models with different parameter value** ***m*** **on training sets (when** ***k1 =*** **0.85,** ***k2 =*** **0.70 and** ***k3*** **= 0.40).** 9A: Sensitivity for the training sets. 9B: Specificity for the training sets.

Using SWCV, 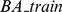 reaches the highest value, 96.18%, using the F-score_SVM, and the optimal parameters of *m*, *k1*, *k2*, *k3*, which are determined by grid searching, are as follows: *m* = 2, *k1 = *0.85, *k2 = *0.70 and *k3* = 0.40. The training accuracies as a function of the parameter *m* were shown in Figure 9A and 9B for the three hybrid models when *k1 = *0.85, *k2 = *0.70 and *k3* = 0.40. As shown in Figure 9, the accuracy rates increase significantly when *m* changes from 1 to 2 for all of the models. For example, the increased rate for F-score_SVM is approximately 5%. In addition, the accuracies of F-score_FDA and F-score_SVM reach a maximum when *m* = 2 except for F-score_BPNN, whose accuracy still increases slightly as *m* varies from 2 to 3. More importantly, the accuracy rates decrease when more than 3 ICs are used in SDA. This result is basically consistent with the report of Lin et al. [53]. Note that the accuracies with *m* = 14 denote the performance without the SDA. For every classification model, those accuracies are distinctly much lower than those when *m* = 2. The results discussed above indicate the remarkable performance of SDA.

Furthermore, Table 3 gives the training accuracies (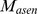,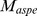) and testing accuracies (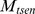,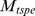) of the six classification models with the optimal grid searching result. First, the accuracy of the model using FDA is obviously lower than the models using BPNN and SVM. This finding suggests that the data from the two types of subjects in the lie detection cannot be separated linearly. Additionally, the performance of the models that use SVM significantly exceeds those of the models that use FDA and BPNN. Using ANOVA, the statistical results (*F*(1, 28)  = 7396.689 and p<0.001) confirm that the testing accuracy for SVM is significantly greater than that for BPNN. The *BA_test* of 96.08% for F-score_SVM strongly suggests that it is suitable for the classification of the two classes of subjects. Additionally, we can see from Table 3 that each hybrid model achieves significantly higher accuracy than the corresponding individual model. For example, on the training sets, SVM reaches a sensitivity and specificity of 91% and 90.98%, respectively. In contrast, F-score_SVM obtains 96.07% and 96.30%, respectively. Based on the above experimental results, the model F-score_SVM reaches the highest classification performance of all of the models.

**Table 3:** Sensitivity/specificity on the training and testing sets for different classification models with the optimal parameter combination.

Classifier models	Sensitivity/specificity (%)	
	Training ( 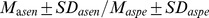 )	Testing ( 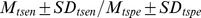 )
FDA	68.38±2.13/67.22±1.94	FDA
BPNN	79.27±1.66/78.78±1.72	BPNN
SVM	91.00±1.80/90.98±1.85	SVM
F-score_FDA	74.65±1.57/74.19±1.70(^▴^)	F-score_FDA
F-score_ BPNN	85.97±1.60/85.60±1.66(*)	F-score_ BPNN

“▴” denotes that a p-value of <0.001 was obtained by ANOVA between F-score_FDA and F-score_SVM; “*” denotes that a p-value of <0.001 was obtained by ANOVA between F-score_BPNN and F-score_SVM; for BPNN, the number of hidden nodes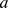 = 5, and the learning rate 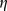  = 0.03; for SVM, radial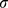 =  32, and penalty parameter *C* = 2^8^.

### Comparison with previous methods

The individual diagnostic rates of the presented and previous methods were calculated, and they were compared in this section. In the BAD/BCD method, each 10 waveforms of each type of response on the Pz electrode were selected to average into a waveform, based on the technique of bootstrapping. In the BAD method, the P300 amplitudes of the three types of responses were calculated based on the Peak-to-Peak method [7], [13], [54]. For the BCD method, the time lag was equal to 0 when the CV was calculated.

**Figure 10:**
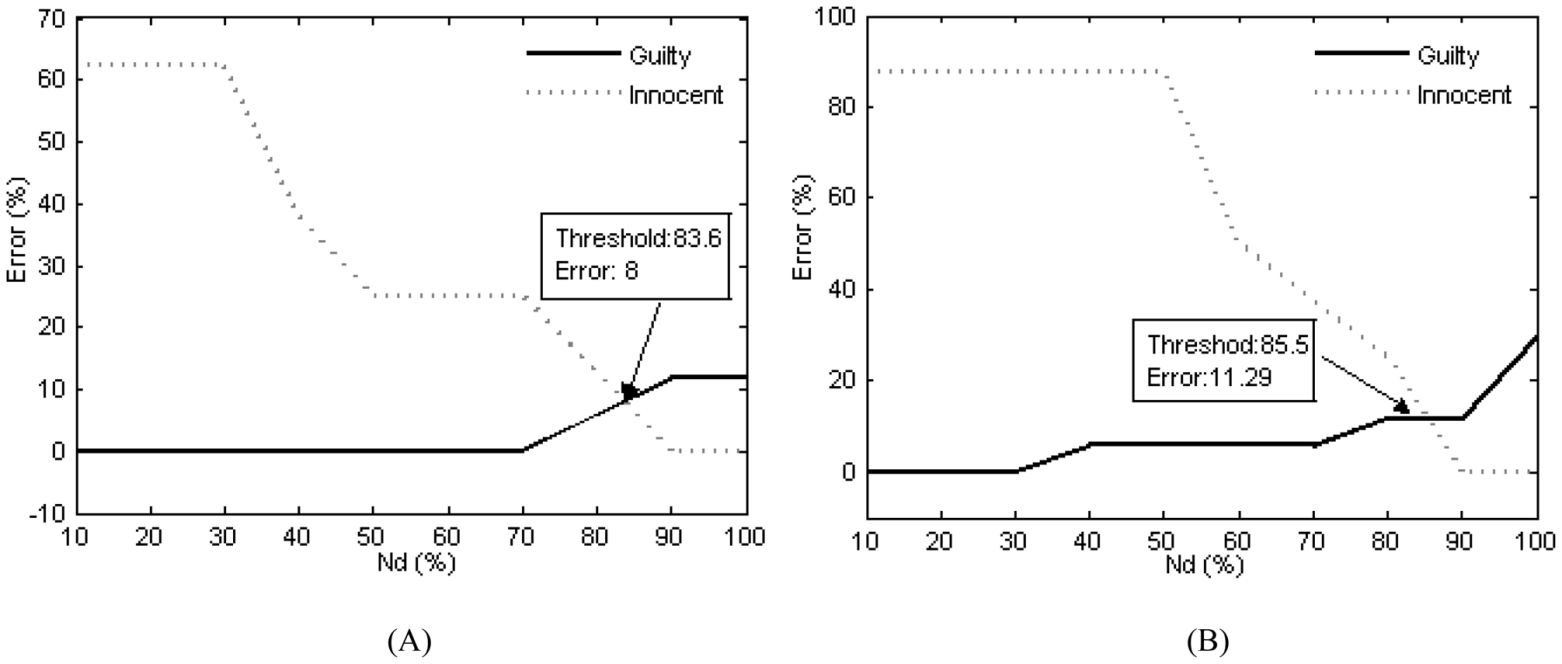
The detection error rates of two groups of subjects. 10A: The detection error rate of the guilty and innocent groups for BAD method. 10B: The detection error rate of the guilty and innocent groups for BCD method.

For the BAD and BCD methods, we calculated 100 *D-*values obtained by 100 iterations for each subject. Let 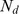 denote the times when the *D-*values were larger than zero. Then 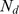 and the percentage of 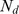 were calculated for each subject, respectively. If the percentage of 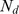 was greater than a threshold 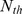, then this subject would be considered to be a guilty subject [7], [12]. Lastly, the error rates of an individual diagnosis as a function of the setting threshold are shown in Figure 10A and 10B, respectively. Considering the equal importance of the detection rates of the two groups of subjects, the individual diagnostic rates of 92% and 88.71% are reached when the thresholds are set to 83.6% and 85.5% for the BAD and BCD methods, respectively.

Based on the results in the above section, for our method, in fact, the individual diagnostic rate can reach 100% when choosing the test accuracy of 90% as a decision criterion for a subject. That is, one was identified as a liar when the percentage of reconstructed samples classified as P300 was larger than 90%. In contrast, one was a truth-teller if the percentage of reconstructed samples classified as *non-P300* was larger than 90%. Obviously, this diagnostic rate is higher than the rates of the BAD and BCD methods, and is also higher than those reported using other machine learning-based methods. For example, Abootalebi et al. [7] reported that the best detection rates are 74%, 80% and 79% for BAD, BCD and the machine learning methods, respectively.

## Discussion and conclusions

Lie detection methods using a large number of stimuli suffer from several inherent drawbacks such as more fatigue for subjects, more workload for examiners, increased probability of countermeasure behavior and lower flexibility [25], [55]. Obviously, a lie detection method with only a small number of stimuli will be crucial for practical lie detection. The purpose of this study is to develop a novel detection method that uses several stimuli to identify the liars, and at the same time, to further increase the individual diagnostic rate and robustness compared to previous studies. For this purpose, we proposed a novel ICA-based SDA to enhance the SNR of P300, and then, we used a machine learning method to distinguish the P300 evoked by guilty subjects from the non-P300 in innocent subjects.

Some recent studies suggested that machine learning-based lie detection methods are more reliable than the BAD and BCD methods. One advantage is that the investigation of the dynamic variation of single trials might help us to study more cognitive information on lying. The second major advantage lies in that the failure of one trial will not affect the classification results of the other trials. In contrast, for BAD and BCD, the failure will change many bootstrapping averages and hence, the overall result of the lie detection [7]. Third, one can utilize more features of P300 in addition to the time-domain features that are used in the BAD/BCD method. Lastly, note that, in previous methods, it is difficult to decide the related thresholds such as the 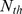 described earlier because this decision involves the tradeoff between the two individual diagnostic rates from the two groups of subjects. In contrast, we can see that this problem does not exist in our method.

In the present study, we assumed that for a P300-based lie detection method, the noise in the single trials could be divided into two categories: one is the ill-assorted responses to a certain type of stimulus, which results from a variation of cognitive state during detection [55]; the other is normal noise such as EOG artifacts and spontaneous EEG. Hence, before applying SAD, we first averaged each 5 raw EEG datasets to decrease the impact of ill-resorted P300′s on the SNR of P300, which would increase the robustness of the entire system for lie detection. The efficiency of this preprocessing method for lie detection is not addressed in this study because it has already been proven in the previous report [55]. To reduce the influence of the second type of noise on the performance of the detection to the greatest extent, we proposed a novel SDA to separate the P300 components from the other noise signals, constructing new Pz waves with the more obvious P300 features; this process can be viewed as a spatial filter for the P300.

Previously, we introduced a topography-template matching (TTM) method [25] to reconstruct P300 waveforms that have a higher SNR. TTM was based on correlation theory of the topography of the ICs. SDA differs from the TTM method in the construction algorithm. SDA is computationally efficient to implement. Hence SDA could decrease the training and testing time. In addition, the classification accuracy of the presented method is higher than that in the report [25]. For the sake of brevity, we have not compared the efficiency of these two methods here and the comparison will be addressed in future studies.

For SDA, the experiment results show that the detection accuracy is the highest when 2 (or 3) P300 ICs are selected to reconstruct the Pz waveform. This finding might indicate that 2 or 3 neural sources are responsible for the task of responding to the P stimuli. This inference deserves further study. In addition, we deemed that the physiology meaning of three parameter values of *k1*, *k2*and *k3* can be interpreted as follows. A realistic P300 IC (unknown P300 independent neural source under scalp) should have different distributed weight on different brain scalp areas. Comparing three *k* values, P300 IC has biggest distributed weight on P3 and P4, medium on Cz and least on Oz scalp areas.

It is worth mentioning that, even though only the waves on the Pz were finally used to extract features, 14 electrodes were still selected to run ICA in order to guarantee the efficiency of the EICA algorithm and SDA. Using ICA has another advantage in that it can help remove the ocular artifacts automatically in the preprocessing phase [24], which few previous studies of lie detection have addressed [56]–[58]. Using SDA to remove ocular artifacts simultaneously will be investigated in the future.

It should be acknowledged that the procedure for tuning parameters in the present study is complicated and time-consuming. However, once these optimal parameter values were selected by the grid searching method on the training sets, they would be kept stable for the testing and real applications. We assumed, for example, that the parameter *m* represents the volume conduction feature of the neurons accounting for the P300 on the scalp, which is thought to be relatively stable spatially [31]. Using other parameter optimization methods [52], [59] is also possible. We will evaluate this approach in future work.

Using the presented method, only 5 Probe stimuli (together with some Target and Irrelevant stimuli) must be presented to the subject in real applications. This arrangement is attractive and promising for practical applications. Moreover, to increase the reliability of the diagnoses, the examiner could perform our testing procedure multiple times and, then, make a more accurate decision by combining several independent testing results.

The F-score, which is a simple feature-selection method, was combined with classifiers to choose the optimal features. The F-score helps to decrease the feature number and, hence, to decrease the computational burden. More importantly, the experimental results show that it helps to enhance the classification accuracy compared with the individual classification models, indicating the importance of the feature selection for the classification performance. For the sake of simplicity, we remove redundant features by a commonly used threshold strategy. In the future, the wrapper method should be used to improve the proposed method.

Different kernel functions for SVM were not tested in this study. It can be found that the training procedure in this study is very complex. Hence, the selection of kernel functions was not considered for the simplicity of the training procedure. In our early other studies [25], [55], we had tested that the radial basis function (RBF) had the best performance than the other kernel functions. Hence, RBF was directly used in SVM method considering the similar lie detection researches.

The proposed method is not specific to research into lie detection and could be extended to other fields of the ERP classification. We believe that more sophisticated feature selection approaches, such as genetic algorithm [7], [60], could further improve the performance of the classifier.

## Supporting Information

File S1 Section S1. FDA classifier. Section S2. BPNN. Section S3. SVM.
(DOC)

## References

[1] Gamer M, Berti S (2010) Task relevance and recognition of concealed information have different influences on electrodermal activity and event-related brain potentials. Psychophysiology 47(2): 355–364. 20003148 10.1111/j.1469-8986.2009.00933.x

[2] Ambach W, Bursch S, Stark R, Vaitl D (2010) A Concealed Information Test with multimodal measurement. Int J Psychophysi 75: 258–26. 10.1016/j.ijpsycho.2009.12.007 20026133

[3] Ito A, Abe N, Fujii T, Ueno A, Koseki Y, et al (2011) The role of the dorsolateral prefrontal cortex in deception when remembering neutral and emotional events. Neurosci Res 69(2): 121–128. 21074583 10.1016/j.neures.2010.11.001

[4] Langleben DD, Loughead JW, Bilker WB, Ruparel K, Childress AR, et al (2005) Telling truth from lie in individual subjects with fast event-related fMRI. Hum Brain Mapp 26(4): 262–272. 16161128 10.1002/hbm.20191 PMC6871667

[5] Phan KL, Magalhaes A, Ziemlewicz TJ, Fitzgerald DA, Green C, et al (2005) Neural correlates of telling lies: a functional magnetic resonance imaging study at 4 Tesla. Acad Radiol 12(2): 164–172. 15721593 10.1016/j.acra.2004.11.023

[6] Rosenfeld JP (2002) Event-related potentials in the detection of deception. Handbook of Polygraph Testing. Academic Press, New York, 265–286.

[7] Abootalebi V, Moradi MH, Khalilzadeh MA (2009) A new approach for EEG feature extraction in P300-based lie detection. Comput Methods and Programs in Biomed 94(1): 48–57. 10.1016/j.cmpb.2008.10.001 19041154

[8] Polich J, Herbst KL (2000) P300 as a clinical assay: rational, evaluation, and findings. Int J Psychophysi 38(1): 3–19. 10.1016/s0167-8760(00)00127-6 11027791

[9] Meijer EH, Smulders FTY, Merckelbach HLGJ, Wolf AG (2007) The P300 is sensitive to concealed face recognition. Int J Psychophysi 66(3): 231–237. 10.1016/j.ijpsycho.2007.08.001 17825933

[10] Rosenfeld JP, Soskins M, Bosh G, Ryan A (2004) Simple, effective countermeasures to P300-based tests of detection of concealed information. Psychophysiology 41(2): 205–219. 15032986 10.1111/j.1469-8986.2004.00158.x

[11] Rosenfeld JP, Labkovsky E, Winograd M. Lui MA, Vandenboom C, et al (2008) The Complex Trial Protocol (CTP): A new, countermeasure-resistant, accurate, P300-based method for detection of concealed information. Psychophysiology 45(6): 906–919. 18823418 10.1111/j.1469-8986.2008.00708.x

[12] Farwell LA, Donchin E (1991) The truth will out: interrogative polygraphy (‘‘lie detection’’) with event-related potentials. Psychophysiology 28(5): 531–547. 1758929 10.1111/j.1469-8986.1991.tb01990.x

[13] Abootalebi V, Moradi MH, Khalilzadeh MA (2006) A comparison of methods for ERP assessment in a P300-based GKT. Int J Psychophysi 62(2): 309–320. 10.1016/j.ijpsycho.2006.05.009 16860894

[14] Dvatzikos C, Ruparel K, Fan Y, Shen DG, Acharyya M, et al (2005) Classifying spatial patterns of brain activity with machine learning methods: Application to lie detection. NeuroImage 28(3): 663–668. 16169252 10.1016/j.neuroimage.2005.08.009

[15] Jung TP, Makeig S, Humphries C, Lee TW, McKeown MJ, et al (2000a) Removing electroencephalographic artifacts by blind source separation. Psychophysiology 37(2): 163–178. 10731767

[16] Wasserman S, Bockenholt U (1989) Bootstrapping: applications to psychophysiology. Psychophysiology 26(2): 208–221. 2727223 10.1111/j.1469-8986.1989.tb03159.x

[17] Jung TP, Makeig S, Waterfield M, Townsend J, Courchesne U, et al (2000b) Removing of eye activity artifacts from visual event-related potentials in normal and clinical subjects. Clin Neurophysiol 111(10): 1745–1758. 11018488 10.1016/s1388-2457(00)00386-2

[18] Bell AJ, Sejnowski TJ (1995) An information-maximization approach to blind separation and blind deconvolution. Neural Computation, MIT Press, Cambridge, MA 7(6): 1129–1159. 10.1162/neco.1995.7.6.1129 7584893

[19] Tang AC, Pearlmutter BA, Zibulevsky M, Carter SA (2000) Blind source separation of multichannel neuromagnetic responses. Neurocomput 32: 1115–1120.

[20] Parra L, Sajda P (2003) Blind source separation via generalized eigenvalue decomposition. J Mach Learn Res 4: 1261–1269.

[21] Peterson DA, Anderson CW (1999) EEG-based Cognitive Task Classification with ICA and Neural Networks. Engineering Applications of Bio-Inspired Artificial Neural Networks. Springer Berlin Heidelberg 1999: 265–272.

[22] Hung CI, Lee PL, Wu YT, Chen LF, Yeh TCH, et al (2005) Recognition of Motor Imagery Electroencephalography Using Independent Component Analysis and Machine Classifiers. Ann Biomed Eng 33(8): 1053–1070. 16133914 10.1007/s10439-005-5772-1

[23] Tang AC, Sutherland MT, Wang Y (2006) Contrasting single-trial ERPs between experimental manipulations: Improving differentiability by blind source separation. NeuroImage 29(1): 335–346. 16256373 10.1016/j.neuroimage.2005.07.058

[24] Gao JF, Yang Y, Lin P, Wang P, Zheng CX (2010) Automatic Removal of Eye-movement and Blink Artifacts from EEG Signals. Brain Topo 23(1): 105–114. 10.1007/s10548-009-0131-4 20039116

[25] Gao JF, Lu L, Yang Y, Yu G, Na LT, et al (2012) A Novel Concealed Information Test Method Based on Independent Component Analysis and Support Vector Machine. Clin EEG Neurosci 43(1): 54–63. 22423552 10.1177/1550059411428715

[26] Comon P (1994) Independent component analysis, a new concept? Signal Process 36(3): 287–314.

[27] Makeig S, Bell AJ, Jung TP, Sejnowski TJ (1996) Independent Component Analysis of Electroencephalgraphic Data. Adv Neural Inform Process Systems 8, MIT press, Cambridge MA, 145––151.

[28] Jung TP, Humphries C, Lee TW, Makeig S, McKeown MJ, et al.. (1998) Extended ica removes artifacts from electroencephalographic recordings. Adv Neural Inform Process Systems, 894–900.

[29] Lee TW, Girolami M, Sejnowski EJ (1999) Independent component analysis using an extended informax algorithm for mixed subgaussian and supergaussian sources. Neural Comput 11(2): 409–433. 10.1162/089976699300016719 9950738

[30] Rosenfeld JP, Ellwanger JW, Nolana K, Wua S, Bermanna RG, et al (1999) P300 Scalp amplitude distribution as an index of deception in a simulated cognitive deficit model. Int J Psychophysi 33(1): 3–19. 10.1016/s0167-8760(99)00021-5 10451015

[31] Xu N, Gao XR, Hong B, Miao XB, Gao SK, et al (2004) BCI Competition 2003—Data Set IIb: Enhancing P300 Wave Detection Using ICA-Based Subspace Projections for BCI Applications. IEEE Trans Biomed Eng 51(6): 1067–1072. 15188880 10.1109/TBME.2004.826699

[32] Polich J (2007) Updating P300: An integrative theory of P3a and P3b. Clin Neurophysiol 118: 2128–2148. 17573239 10.1016/j.clinph.2007.04.019 PMC2715154

[33] Demiralp T, Ademoglu A, Schurmann M, Eroglu CB, Basar E (1999) Detection of P300 waves in single trials by the Wavelet Transform (WT). Brain Lang 66(1): 108–128. 10080867 10.1006/brln.1998.2027

[34] Kalatzis I, Piliouras N, Ventouras E, Papageorgiou CC, Rabavilas AD, et al (2004) Design and implementation of an SVM-based computer classification system for discriminating depressive patients from healthy controls using the P600 component of ERP signals, Comput Meth Prog Biomed. 75(1): 11–22. 10.1016/j.cmpb.2003.09.003 15158043

[35] Hsu WY, Lin CC, Ju MS, Sun YN (2007) Wavelet-based fractal features with active segment selection: Application to single-trial EEG data. J Neurosci Meth 163(1): 145–160. 10.1016/j.jneumeth.2007.02.004 17379316

[36] Herrmann CS, Knight RT (2001) Mechanisms of human attention: event-related potentials and oscillations. Neurosci and Biobehav Rev 25(6): 465–476. 11595268 10.1016/s0149-7634(01)00027-6

[37] Yong YPA, Hurley NJ, Silvestre GCM (2005) Single-trial EEG classification for brain-computer interface using wavelet decomposition. Eur Signal Process.

[38] Mrzagora AC, Bunce S, Izzetoglu M, Onaral B (2006) Wavelet analysis for EEG feature extraction in deception detection Proceedings of the 28th IEEE EMBS Annual International Conference. New York City, USA, Aug 30. 10.1109/IEMBS.2006.260247 17946114

[39] Ademoglu A, Micheli-Tzanakou E, Istefanopulos Y (1997) Analysis of pattern reversal visual evoked potentials (PRVEPs) by spline wavelets. IEEE Trans on Biomed Eng 44(9): 881–890. 10.1109/10.623057 9282480

[40] Unser M, Aldroubi A, Eden M (1992) On the asymptotic convergence of B-spline wavelets to Gabor functions. IEEE Trans on Information Theory 38(2): 864–872.

[41] Quiroga RQ, Sakowitz OW, Basar E, Schurmann M (2001) Wavelet transform in the analysis of the frequency composition of evoked potentials. Brain Res Protoc 8(1): 16–24. 10.1016/s1385-299x(01)00077-0 11522524

[42] Chen FL, Li FC (2010) Combination of feature selection approaches with SVM in credit scoring. Expert Syst Appl 37: 4902–4909.

[43] Polat K, Güneş S (2009) A new feature selection method on classification of medical datasets: Kernel F-score feature selection. Expert Syst Appl 36(7): 10367–10373.

[44] Jouve PE, Nicoloyannis N (2005) A filter feature selection method for clustering Foundations of Intelligent Systems. Springer Berlin Heidelberg, 583–593.

[45] Kohavi R, John GH (1997) Wrappers for feature subset selection. Arti Intell 97(1): 273–324.

[46] Huang CJ, Dian X, Chuang YT (2007) Application of wrapper approach and composite classifier to the stock trend prediction. Expert Syst Appl 34(4): 2870–2878.

[47] Chiang L, Russell E, Braatz R (2000) Fault diagnosis in chemical processes using Fisher discriminant analysis, discriminant partial least squares, and principal component analysis. Chemomet Intell Lab Syst 50(2): 243–252.

[48] Tarassenko L, Khan YU, Holt MRG (1998) Identification of inter-ictal spikes in the EEG using neural network analysis. IEE Proceedings Science, Measurement & Technology 145(6): 270–278.

[49] Kaper M, Meinicke P, Grossekathoefer U, Lingner T, Ritter H (2004) BCI competition 2003-data set IIb: support vector machines for the P300 speller paradigm, IEEE Trans on Biomed Eng. 51(6): 1073–1076. 10.1109/TBME.2004.826698 15188881

[50] Shoker L, Sanei S, Chambers J (2005) Artifact removal from electroencephalograms using a hybrid BSS-SVM algorithm. IEEE Sig Process Letters 12(10): 721–724.

[51] Shao SY, Shen KQ, On CJ, Wilder-Smith EPV, Li XP (2009) Automatic EEG artifact removal: A weighted support vector machine approach with error correction. IEEE Trans Biomed Eng 56(2): 336–344. 19272915 10.1109/TBME.2008.2005969

[52] Burges C (1998) A tutorial on support vector machines for pattern recognition. Data Mining and Knowl Discov 2(2): 121–167.

[53] Lin CT, Chung IF, Ko LW, Chen YC, Liang SF, et al (2007) EEG-Based Assessment of Driver Cognitive Responses in a Dynamic Virtual-Reality Driving Environment. IEEE Trans Biomed Eng 54(7): 1394–1352. 10.1109/TBME.2007.891164 17605367

[54] Soskins M, Rosenfeld JP, Niendam T (2001) The case for peak-to-peak measurement of P300 recorded at.3 Hz high pass filter settings in detection of deception. Int J Psychophysi 40(17): 173–1800. 10.1016/s0167-8760(00)00154-9 11165356

[55] Gao JF, Yan XG, Sun JC, Zheng CX (2011) Denoised P300 and Machine Learning-based Concealed Information Test Method. Comput Meth Prog Bio 104: 410–417. 10.1016/j.cmpb.2010.10.002 21126796

[56] Matsuda I, Nittono H, Hirota A, Ogawa T, Takasawa N (2009) Event-related brain potentials during the standard autonomic-based concealed information test. Int J Psychophysi 74(1): 58–68. 10.1016/j.ijpsycho.2009.07.004 19631702

[57] Matsuda I, Nittono H, Ogawa T (2011) Event-related potentials increase the discrimination performance of the autonomic-based concealed information test. Psychophysiology 48(12): 1701–1710. 21806637 10.1111/j.1469-8986.2011.01266.x

[58] Matsuda I, Nittono H, Ogawa T (2013) Identifying concealment-related responses in the concealed information test. Psychophysiology 50: 617–626. 23560794 10.1111/psyp.12046

[59] Friedrichs F, lgel C (2005) Evolutionary tuning of multiple SVM parameters. Neurocomput 24: 107–117.

[60] Wu CH, Tzeng GH, Lin RH (2009) A novel hybrid genetic algorithm for kernel function and parameter optimization in support vector regression. Expert Syst Appl 36: 4725–4735.

