# Supplementary material for: A Novel Algorithm to Enhance P300 in Single Trials: Application to Lie Detection Using F-Score and SVM
Source: PLoS One. 2014 Nov 3;9(11):e109700. doi: 10.1371/journal.pone.0109700 (PMC4218862; doi:10.1371/journal.pone.0109700)
Supplement: File S1 — Section S1. FDA classifier. Section S2. BPNN. Section S3. SVM. (DOC) [file pone.0109700.s001.doc]

**S1. FDA classifier**

For a classification problem of two classes of samples$\omega_{1}$and$\omega_{2}$, FDA aims at seeking a vector $\overrightarrow{\mathbf{W}}$ on which the projection of $\omega_{1}$and$\omega_{2}$ can be separated from each other, meanwhile, the cluster variance, calculated by the projection of $\omega_{1}$and$\omega_{2}$, maintains to the lowest extent. Suppose $\mathbf{S}_{b}=(\overrightarrow{m_{1}}-\overrightarrow{m_{2}})(\overrightarrow{m_{1}}-\overrightarrow{m_{2}})^{'}$ denotes the between-class scatter matrix, and $\mathbf{S}_{w}=\sum_{\overrightarrow{x}\in\omega_{1}} (\overrightarrow{x}-\overrightarrow{m_{1}})(\overrightarrow{x}-\overrightarrow{m_{1}})^{'}+\sum_{\overrightarrow{x}\in\omega_{2}} (\overrightarrow{x}-\overrightarrow{m_{2}})(\overrightarrow{x}-\overrightarrow{m_{2}})^{'}$ denotes the within-class scatter matrix, where $\overrightarrow{m_{1}}$and $\overrightarrow{m_{2}}$ denote the mean vectors of two classes of samples, respectively. By maximizing

$J(\overrightarrow{\mathbf{W}})=\frac{\overrightarrow{\mathbf{W}}^{'}S_{b}\overrightarrow{\mathbf{W}}}{\overrightarrow{\mathbf{W}}^{'}S_{w}\overrightarrow{\mathbf{W}}}$ (A.1)

$\overrightarrow{\mathbf{W}}$ can be obtained. In practical applications, this maximization problem was solved by decomposition of eigenvalue. In this study, when testing a sample, its class label was determined by simply comparing two projected distance values between the $\overrightarrow{x}$ and the two mean vectors.

**S2. BPNN**

In this study, the network consisted of an input layer, a hidden layer and an output layer. The number of the input node corresponded to the dimension of every sample, and there was only one node in the output layer. The training was accomplished by iterating two phases: forward computation and backward adjust of error. First, the network output at *n*th iteration can be expressed as follows:

$$o(n)=F(\sum_{j=1}^{a} W_{j}(n)y_{j}(n))$$

$=F(\sum_{j=1}^{a} W_{j}(n)(\sum_{i=1}^{b} x_{i}(n)U_{ij}(n)))$ (A.2)

where $U_{ij}$ denotes weight connecting *i*th input node to *j*th hidden node; $a$ denotes number of the hidden node; $b$denotes number of the input node; $W_{j}$denotes weight connecting *j*th hidden node to output node and *F* denotes the output mapping function such as sigmoid function. When$e(n)$, the difference between real output and desired output, was greater than a control precision$\varepsilon$, backward adjust procedure was used to adjust weight $W_{j}(n)$ as followings:

$W_{j}(n+1)=W_{j}(n)+\eta\delta(n)y_{j}(n)$ (A.3)

where $\delta(n)y_{j}(n)=[e(n)(1-o(n))o(n)]y_{j}(n)$ is local gradient of the output layer and $\eta$ is learning rate. In this study, when testing a sample, its class label was determined by the network output.

**S3. SVM**

The goal of SVM is to find an optimal hyperplane that maximizes the separating margin between $\omega_{1}$and$\omega_{2}$. It can be solved by the following minimization procedure with a constraint condition:

$$\min(C\sum_{i=1}^{k} \gamma_{j}+\frac{1}{2}\left\|\overrightarrow{W}\right\|^{2})$$

s.t. , i = 1, 2, …, *k* (A.4)

where $l_{i}\in\left\{\pm1\right\}$ is the class label of the *i*th sample with *k* being the number of SV. $g_{i}$ denotes the feature vector of the *i*th sample; $\overrightarrow{W}$and *b* denotes the orientation and offset of the hyperplane, respectively. $\left\|\overrightarrow{W}\right\|^{2}$ is used to calculate the squared Euclidean norm, denotes the dot product. $\gamma_{i}$ is called slack parameter. C is a penalty factor and can be determined by the cross validation procedure. Above optimization problem can be solved by introducing the other optimization for Lagrangian multipliers $\alpha_{i}$(Shao et al., 2009). A sample $g_{i}$ is a Support vectors (SV) when it corresponds to a nonzero$\alpha_{i}$. Let $g_{i}^{s}$ denote a SV, and then the class label of any test sample g can be given as followings:

$l(g)=\mathrm{sgn}(\sum_{i=1}^{k_{s}} l_{i}\alpha_{i}K(g_{i}^{s},g)+b)$ (A.5)

where $K_{s}$ is the number of SV. “$\mathrm{sgn}$” denotes a flag function which transforms a negative input value to -1 and positive value to 1. Notation *K* denotes a kernel function which is used to project the samples to a new feature space with a higher dimension where the samples can be linearly separated. In this study, related parameter values in the kernel function were also determined by cross validation procedure.
